# Red-Emissive Sulfur-Doped Carbon Dots for Selective and Sensitive Detection of Mercury (II) Ion and Glutathione

**DOI:** 10.3390/ijms23169213

**Published:** 2022-08-17

**Authors:** Jinjin Zeng, Linhong Liao, Xiao Lin, Genyan Liu, Xiaogang Luo, Ming Luo, Fengshou Wu

**Affiliations:** 1 Hubei Key Laboratory of Novel Reactor and Green Chemical Technology, School of Chemical Engineering and Pharmacy, Wuhan Institute of Technology, Wuhan 430072, China; 2 Key Laboratory of Novel Biomass-Based Environmental and Energy Materials in Petroleum and Chemical Industry, Key Laboratory for Green Chemical Process of Ministry of Education, Wuhan Institute of Technology, Wuhan 430072, China; 3 School of Materials Science and Engineering, Zhengzhou University, Zhengzhou 450001, China; 4 School of Materials Science and Engineering, Wuhan University of Technology, Wuhan 430070, China

**Keywords:** carbon dots, red emission, Hg^2+^, glutathione, bioimaging

## Abstract

Carbon dots (CDs) show great potential in bioimaging and biosensing because of their good biocompatibility and excellent optical properties. However, CDs with intense red emissions for sensitive and selective detection are rarely reported. Herein, we prepared the red-emissive carbon dots (RCDs) through a facile hydrothermal method using tetra (4-carboxyphenyl) porphyrin (TCPP) and thiourea as starting materials. The obtained RCDs were characterized by TEM, XRD, and XPS. RCDs exhibited high water solubility and strong red emission (λ_em_ = 650 nm), with the fluorescence quantum yield as high as 26.7%, which was greatly higher than that of TCPP. Moreover, the as-prepared RCDs could be acted as a highly selective and sensitive probe for the detection of Hg^2+^ and glutathione (GSH) through the fluorometric titration method. The detection limits of Hg^2+^ and GSH were calculated to be 1.73 and 1.6 nM, respectively. The cellular experiments demonstrated the good biocompatibility of RCDs and their feasibility in bioimaging. Thus, this work provided a simple strategy to design and synthesize the highly red-emissive carbon dots, which showed promising application in biological and environmental assays.

## 1. Introduction

As a new type of carbon-based nanomaterials, carbon dots (CDs) with particle size less than 10 nm [1], had drawn considerable interest owing to their distinct properties such as high water solubility [2], excellent biocompatibility [3], and remarkable optical properties [4,5]. Since they were first discovered in 2004 [6], most studies had focused on the synthetic methods and biomedical applications of CDs. However, the traditional method for preparing CDs usually emitted blue or green fluorescence, which was easily affected by the auto-fluorescence of the biological tissues [7,8,9]. Moreover, lights with short wavelengths are harmful to tissues and cells due to their high energy. Thus, CDs bearing blue or green fluorescence were greatly limited in biomedical applications [10,11]. In contrast, the red or near-infrared-emissive CDs (RCDs) showed great potential in the biomedical field because of their deep tissue penetration ability [12,13,14,15]. Therefore, it is highly desirable to develop new methods to fabricate RCDs for multifunctional applications, such as bioimaging, biosensing, and cancer theranostic. Furthermore, the surface of carbon dots was full of oxygen-containing groups, which could strongly interact with metal ions, resulting in fluorescence quenching. Hg (II) is one of the most toxic heavy metals in the environment, which seriously threatens propagation growth and human health. Therefore, the detection and quantification of Hg (II) are of great significance for environmental monitoring and protection. Since Hg (II) showed a high affinity toward sulfur elements, the sulfur-doped CDs might be acted as a suitable fluorescence sensor for Hg^2+^.

On the other hand, porphyrin and its derivatives, which bear a tetrapyrrole skeleton, had attracted great attention in the field of photoelectronics [16], catalysis [17], bioimaging [18], and photodynamic therapy [1], because of their easy functionalization [19], high stability [20], and remarkable photochemical properties [21]. Additionally, the porphyrin molecules were demonstrated as promising photosensitizers for photodynamic therapy and fluorescence detection because of their ability to generate reactive oxygen species and red emission simultaneously under light irradiation [22,23,24]. However, the extreme hydrophobicity and low fluorescence quantum yield of porphyrins greatly limited their applications in bioimaging and cancer therapy [25,26]. Considering the large π-π conjugated structure [27], high thermal stability [28], and red fluorescence of porphyrin molecules [29], the porphyrins with functional groups, such as hydroxyl, carboxyl, and amino groups, should be the potential precursors for the preparation of red-emissive RCDs.

**Scheme 1:**
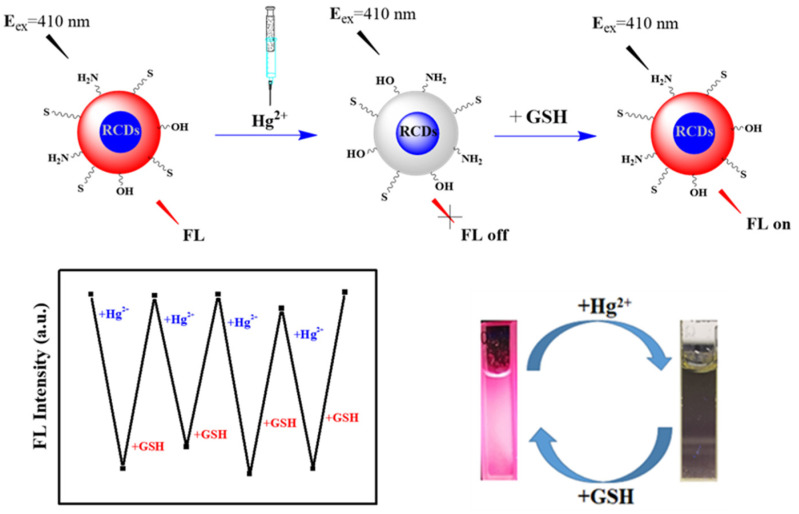
The selective sensing process of RCDs.

In this context, here, we prepared the red-emissive carbon dots through a one-pot hydrothermal method with tetra (4-carboxyphenyl) porphyrin (TCPP) and thiourea as precursors. The structure and morphology of RCDs were confirmed by transmission electron microscopy (TEM), X-ray photoelectron spectroscopy (XPS), X-ray diffraction (XRD), and Fourier transform infrared (FT-IR). The as-prepared RCDs exhibited strong red emission (λ_em_ = 650 nm) with fluorescence quantum yield as high as 26.7%, which was greatly higher than that of porphyrin precursor and previously reported RCDs [30,31,32]. Most importantly, the fluorescence of RCDs could be quenched with the addition of Hg^2+^, probably due to the presence of sulfur elements on the surface of RCDs. Moreover, the fluorescence of the RCDs/Hg^2+^ system would be completely restored after the addition of glutathione (GSH) and this process could be repeated multiple times without any variation, indicating the high accuracy of this method. The detection limits of Hg^2+^ and GSH based on this fluorometric titration method were measured to be 1.73 and 1.6 nM, respectively. Moreover, the biocompatibility and bioimaging application of RCDs were verified through the MTT method and laser scanning confocal microscope, respectively. Thus, this work provided a new strategy to develop strongly red-emissive carbon dots, which could serve as a selective and sensitive FL probe for Hg^2+^ and GSH detection (Scheme 1). The as-prepared single platform for multiple detection was highly desirable because of the advantages of convenience and simplicity.

## 2. Results and Discussion

### 2.1. Synthesis of RCDs

**Scheme 2:**
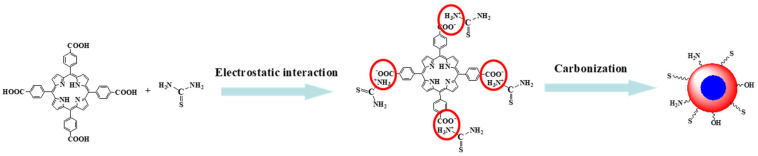
Schematic illustration of the proposed synthetic procedures of RCDs.

RCDs were fabricated through a one-step hydrothermal method with TCPP and thiourea as starting materials. Specifically, the TCPP was first dispersed into the aqueous solution of thiourea under ultrasound conditions, whereupon the carboxyl groups on TCPP were surrounded by thiourea through electrostatic interaction to form the polymer-like CDs. When the temperature was higher than 180 °C (Figure S1), the formed polymers would gradually decompose, followed by the carbonization to form the carbon dots (Scheme 2).

### 2.2. Characterization of RCDs

**Figure 1:**
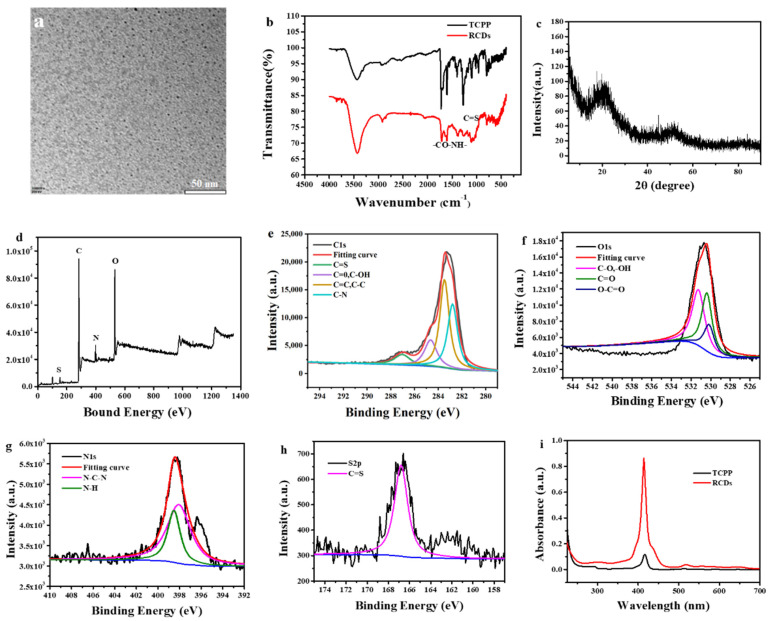
(**a**) TEM image of RCDs; (**b**) FT-IR spectra of TCPP and RCDs; (**c**) XRD curve of RCDs; (**d**) XPS spectra of RCDs; (**e**) High-resolution spectrum of C1s; (**f**) O1s; (**g**) N1s and (**h**) S2p of CDs; (**i**) UV-vis absorption spectra of RCDs and TCPP.

RCDs displayed good spherical morphology and uniform particle size distribution from 4 to 10 nm, as shown in the TEM image (Figure 1a). To further verify the particle size distribution, the DLS of RCDs was measured. As indicated in Figure S2, the particle size of RCDs ranged from 3 to 9 nm with an average particle size of 5.6 nm, which agreed well with the result of TEM data. To verify the success of the reaction between TCPP and thiourea, FT-IR analysis was performed (Figure 1b). The typical absorption band at 3100–3500 cm^−1^ could be assigned to the stretching vibrations of O-H and N-H groups. These bands suggested that the hydroxyl and amide groups were the main functional groups on the surface of RCDs, contributing to their high solubility in the aqueous solution. The peak at 1714 cm^−1^ was assigned to the stretching vibrations of amide linkage (-CONH), indicating the success of the reaction between TCPP and thiourea. Compared to that of TCPP, the absorption band at 1084 cm^−1^ was observed in RCDs, which could be assigned to the stretching vibration of C = S. Thus, the sulfur element was successfully doped in RCDs. The XRD pattern of RCDs showed wide diffraction peaks between 20° and 25° (Figure 1c), probably attributed to the amorphous structure of carbon-based materials [33].

The chemical composition of RCDs was verified through X-ray photoelectron spectroscopy. As shown in Figure 1d, RCDs mainly consisted of C, O, N, and S elements. The XPS survey spectra of RCDs showed four peaks at 532 eV, 397 eV, 282 eV, and 153 eV, corresponding to O1s, N1s, C1s, and S2p, respectively. The C1s XPS spectrum of RCDs can be decomposed into four peaks centered at 287.2 eV (C = S), 284.7eV (C = O/C-OH), 283.5 eV (C = C/C-C), and 282.8 eV (C-N), respectively (Figure 1e) [34]. The XPS spectrum of O1s was decomposed into three peaks at 530.2 eV (O-C = O), 530.4 eV (C = O), and 531.3 eV (C-O/-OH) (Figure 1f) [35]. The detailed information on N1s was shown in Figure 1g, which was split into two peaks at 398 eV (N-C-N) and 398.6 eV (N-H), respectively [36]. The high-resolution spectrum of S2p (Figure 1h) revealed the presence of the C = S bond (166.8 eV) in RCDs [37]. These results further confirmed the success of S element doping in RCDs. To further verify the stability of RCDs, the size of RCDs in the buffer (pH = 6) was monitored for 5 days using DLS. As indicated in Figure S3, no significant variation of size was found for RCDs, indicating their high stability in vitro.

### 2.3. Optical Properties of RCDs

The absorption spectra of TCPP and RCDs were measured in dichloromethane and deionized water, respectively. As indicated in Figure 1i, RCDs exhibited a weak peak around 300 nm, which was probably ascribed to the n-π* transition [38]. Moreover, an intense absorption peak at 415 nm was found in RCDs, which was consistent with the characteristic Soret band of the porphyrin molecule [39], indicating the parent structure of porphyrin was preserved after the hydrothermal reaction.

**Figure 2:**
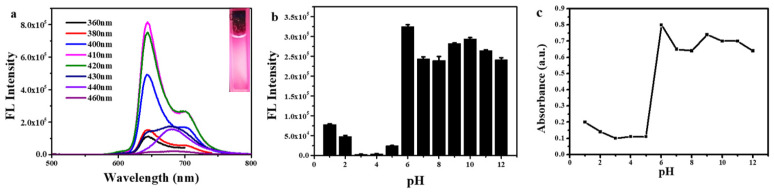
(**a**) Fluorescence spectra of RCDs in water (the inset is the photograph of RCDs under UV lamp (365 nm) excitation); (**b**) FL intensity of RCDs at different pH values; (**c**) The absorbance of RCDs at 415 nm under different pH conditions.

RCDs emitted strong red fluorescence in the aqueous solution under irradiation of a 365 nm UV lamp (see the inset of Figure 2a). To investigate the optical properties of RCDs, the emission spectra of RCDs were recorded under different excitation wavelengths ranging from 360 to 460 nm. As shown in Figure 2a, the fluorescence spectra of RCDs did not show typical excitation-dependent emission behavior as reported in other CDs [40]. The maximum emission peak of RCDs was located at around 650 nm when excited at 410 nm, which was consistent with the related excitation spectra (Figure S4). Using Rhodamine B (QY = 89%) as a standard, the fluorescence quantum yield of RCDs was calculated to be 26.7%.

The pH value was a key factor that affected the fluorescence of carbon dots [41]. As shown in Figure 2b, the FL intensity of RCDs exhibited pH-dependent properties. RCDs displayed the strong FL intensity under weak acid and alkaline conditions (pH = 6–12). However, the FL intensity of RCDs dramatically decreased with the pH value ranging from 5 to 1. The absorption spectrum of RCDs at different pH values was further measured (Figure 2c), where the absorbance at 415 nm decreased significantly in acid solution compared to that in neutral and alkaline conditions, showing a similar tendency to that of the fluorescence spectrum.

### 2.4. FL On-Off of RCDs in the Presence of Hg (II) and Possible Mechanism

**Figure 3:**
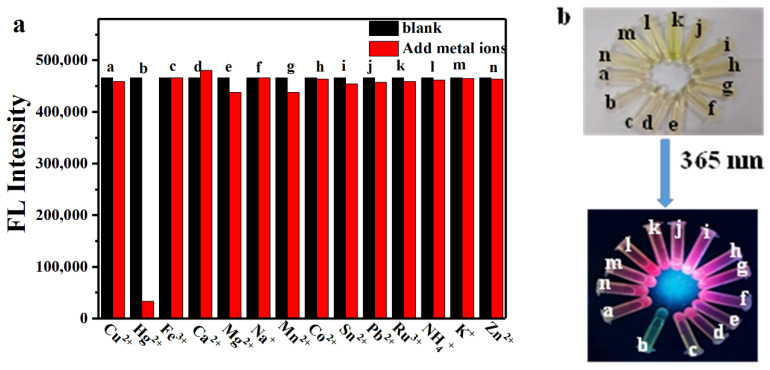
(**a**) Selectivity evaluation of RCDs-based sensor for different ions in PBS solution (pH = 6); (**b**) Visible emission of RCDs upon the addition of different ions under UV light irradiation (365 nm) (a: Cu^2+^, b: Hg^2+^, c: Fe^3+^, d: Ca^2+^, e: Mg^2+^, f: Na^+^, g: Mn^2+^, h: Co^2+^, i: Sn^2+^, j: Pb^2+^, k: Ru^3+^, l: NH_4_^+^, m: K^+^, n: Zn^2+^).

In general, the oxygen functional groups on the surface of CDs contributed not only to water solubility but also to their strong interaction with metal ions [42]. Accordingly, the effects of 14 common cations, including Cu^2+^, Fe^3+^, Co^2+^, Mn^2+^, Na^+^, K^+^, Mg^2+^, Ca^2+^, Zn^2+^, Hg^2+^, Pb^2+^, NH_4_^+^ Sn^2+^ and Ru^3+^, on the FL response of RCDs were investigated. As shown in Figure 3a, the fluorescence of RCDs was obviously quenched with the addition of Hg^2+^, while other cations had no significant effects on their FL intensity. The fluorescence quenching effect of cations was further evidenced by comparing with the different RCDs/ion systems under UV light (365 nm) irradiation (Figure 3b). The selectivity of RCDs towards Hg^2+^ was probably attributed to the high affinity between Hg^2+^ and sulfur element on the surface of RCDs. Furthermore, the oxygen-containing groups on the surface of carbon dots could interact strongly with Hg^2+^, forming the complex of CDs-Hg^2+^. Thus, these synergistic effects might contribute to the fluorescence quenching of RCDs.

**Figure 4:**
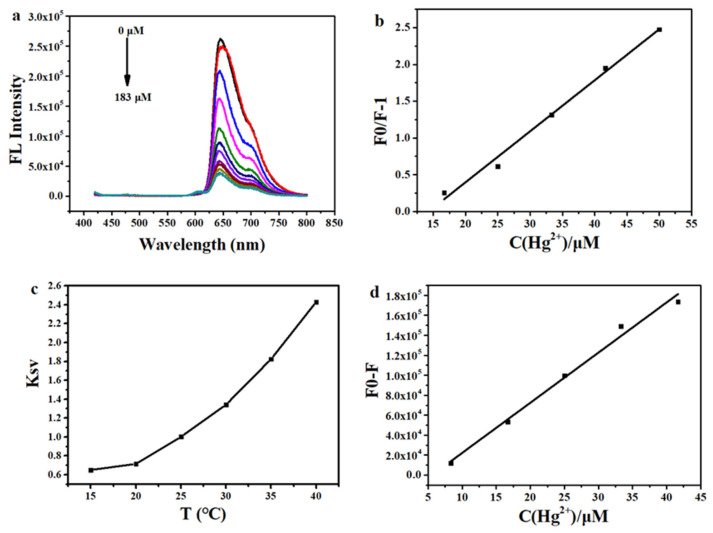
(**a**) Effect of Hg^2+^ concentration on the fluorescence intensity of RCDs; (**b**) Effect of Hg^2+^ concentrations on F0/F − 1; (**c**) The change of Stern-Volmer quenching constant (Ksv) with the temperature; (**d**) Effect of Hg^2+^ concentrations on F0-F.

To ensure the proposed application in the sensitive detection of Hg^2+^, the detection limit of the sensing system was studied. As indicated in Figure 4a, the fluorescence intensity of RCDs decreased gradually with the addition of Hg^2+^ from 0 to 183 μM. When the concentration of Hg^2+^ was in the low range (5–50 μM), the linear relationship between Hg^2+^ concentration and fluorescence intensity was obtained (Figure 4b), which followed the Stern-Volmer equation (F0/F = 1 + Ksv[C], where Ksv was Stern–Volmer quenching constant, [C] was Hg^2+^ concentration, and F0 and F were FL intensity of RCDs at 650 nm) [43]. As shown in Figure 4c, the Stern–Volmer quenching constant (Ksv) increased with the temperature, indicating a dynamic quenching process. Moreover, the linear regression equation was obtained as ΔF = F0 − F = a + b[C] with a correlation coefficient of 0.9918, where F0 was the blank fluorescence intensity, F was the fluorescence intensity after the addition of Hg^2+^, [C] was the concentration of Hg^2+^, and a and b were constants. According to this linear regression equation (Figure 4d), the detection limit was determined as 1.73 nM (S/N = 3). Thus, RCDs could be used as a selective and sensitive fluorescence sensor for the assay of Hg^2+^.

### 2.5. FL Restoration of RCDs/Hg (II) in the Presence of GSH and Possible Mechanism

**Figure 5:**
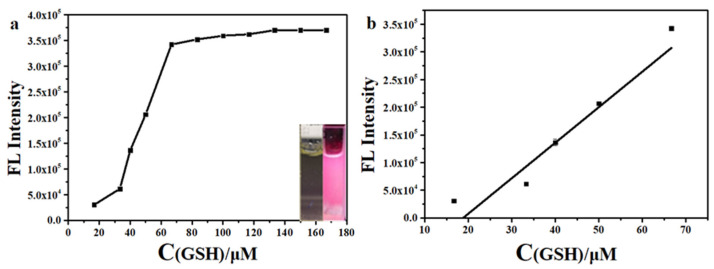
(**a**) Fluorescence intensity (λ_ex_ = 410 nm) of the RCDs/Hg^2+^ system upon the addition of GSH in PBS buffer (pH = 6) (the insert was the emission of the system under UV light irradiation before and after GSH addition); (**b**) The linear correlation between fluorescence intensity and GSH concentration.

Since thiol-containing glutathione (GSH) had a high affinity towards Hg^2+^, it might recover the quenched fluorescence of the RCDs/Hg^2+^ system. To verify this inference, the fluorescence spectrum of the RCDs/Hg^2+^ system was recorded with the addition of GSH. As shown in Figure 5a, the quenched fluorescence of RCDs/Hg^2+^ was gradually recovered with the GSH concentration increasing from 0 to 65 µM, which was probably ascribed to the release of Hg^2+^ from the surface of RCDs through the formation of a strong Hg (II)-GSH complex. The recovered fluorescence intensity of RCDs displayed a good linear relationship between F and the GSH concentration ranging from 0 to 65 µM (Figure 5b), where F was the fluorescence intensity of the RCDs/Hg^2+^ system in the presence of GSH. Based on this correlation, the detection limit of GSH was calculated to be 1.6 nM.

**Figure 6:**
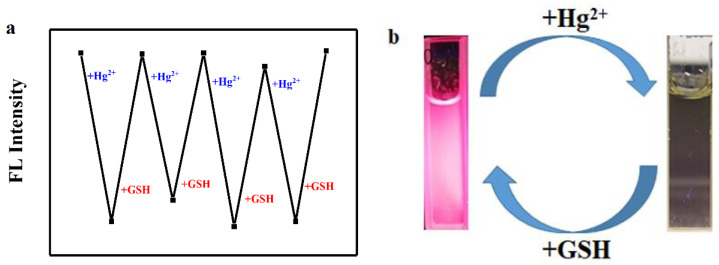
(**a**) The fluorescence intensity of RCDs after the addition of Hg^2+^ and GSH for multiple cycles and (**b**) the related emission change under 365 nm lamp irradiation.

The reversibility of RCDs as the selective fluorescence sensor towards Hg^2+^ and GSH was evaluated by recording the fluorescence change of the system during the multiple cycles (Figure 6). As shown in Figure 6a, the red fluorescence of RCDs went through off-on and on-off process upon the addition of Hg^2+^, GSH, and Hg^2+^ successively, further confirming that RCDs could be dissociated from the RCDs/Hg^2+^ system upon the addition of GSH. Accordingly, the dissociated RCDs in the system could be reused as a fluorescence sensor for Hg^2+^ in the next cycle. Moreover, the fluorescence intensity of RCDs could return to the original level after multiple cycles (Figure 6b), indicating the high stability of this sensing system based on RCDs.

### 2.6. Selectivity of the RCDs/Hg (II) System and Interference Measurements

**Figure 7:**
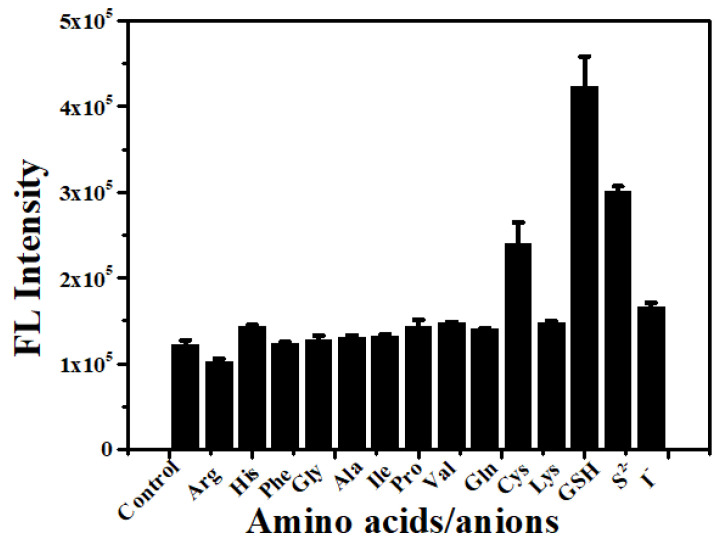
Fluorescence intensity (λ_ex_ = 410 nm) of the RCDs/Hg^2+^ system (control) after the addition of different amino acids and anions (the concentration of the amino acids and anions is 500 µM).

To evaluate the selectivity of the RCDs/Hg^2+^ system for GSH detection, the fluorescence change upon the addition of other amino acids and anions (S^2−^ and I^−^) was investigated. As shown in Figure 7, besides Cys and S^2−^, the addition of other amino acids and anions had no significant effect on the fluorescence of the RCDs/Hg^2+^ system. In addition, the FL recovery efficiency of GSH was significantly higher than that of Cys and S^2−^, indicating RCD could be used as a highly selective platform for the detection of GSH through a facile fluorometric method.

### 2.7. Cytotoxicity Assay of RCDs

**Figure 8:**
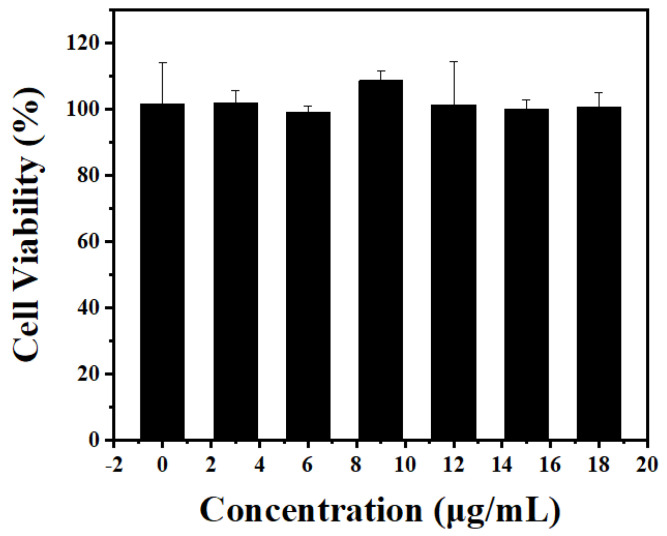
Viability of Hepa 1-6 cells at different concentrations (0, 3, 6, 9, 12, 15 and 18 μg/mL) of RCDs (n = 5, *p* < 0.05 for each group).

The biocompatibility of RCDs was studied using the standard MTT method against tumorigenic liver (Hepa 1-6) cells. As shown in Figure 8, the tumorigenic liver cells that were incubated with RCDs maintained high viability (>90%) even at a high concentration, suggesting the good biocompatibility of RCDs.

### 2.8. Bioimaging of RCDs

**Figure 9:**
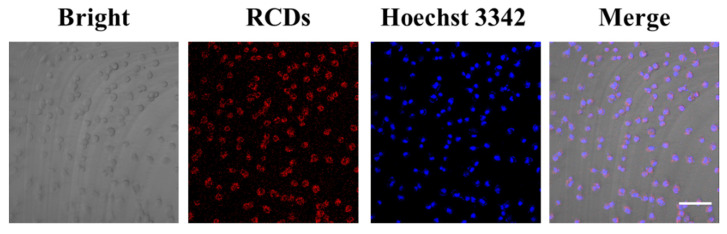
Laser scanning confocal microscope image of Hepa 1-6 cells incubated with RCDs. Nuclei (blue) were stained with Hoechst 33,342 (50 μM) (scale bar = 25 μm).

Considering the good biocompatibility of RCDs, we further conducted the application of RCDs in bioimaging through the laser scanning confocal microscope image. As shown in Figure 9, the Hepa 1-6 cells that incubated with RCDs exhibited intense red fluorescence in the cytoplasm, suggesting the efficient accumulation of RCDs in cancer cells.

## 3. Materials and Methods

### 3.1. Materials

Tetra(4-carboxyphenyl)porphyrin (TCPP) was synthesized according to the method that was described in the previous literature [44]. Thiourea, DMF, MnCl_2_, CaCl_2_, Mg(OAc)_2_, NiCl_2_, CoCl_2_, CuCl_2_, FeCl_3_, HgCl_2_, Zn(OAc)_2_, Na_2_S, NaI, L-glutathione (GSH), L-cysteine (Cys), L-arginine (Arg), β-alanine (Ala), L-lysine (Lys), glycine (Gly), L-histidine (His), L-glutamine (Gln), L-isoleucine (lle), L-phenylalanine (Phe), L-proline (Pro) and L-valine (Val) were purchased from Innochem (Beijing, China), and used without further purification.

### 3.2. Characterization

The Fourier transform infrared spectra (FT-IR) was obtained by NICOLET380 FT-IR spectrometer (PerkinElmer, Waltham, MA, USA) using the KBr disk method. The absorption spectrum was performed on a UV-vis spectrophotometer (UV-3200PC) (Shanghai, China). The fluorescence spectrum was recorded by an FL spectrophotometer (Varian, Inc., Palo Alto, Santa Clara, CA, USA). The powder X-ray diffraction (XRD) pattern was obtained through graphite monochromatized Cu Kα radiation (D8ADVANCEDaVinci, Bruker, Shanghai, China). The Transmission electron microscope (TEM) image was recorded by the Tecnai G2 F30 S-Twin of Philips FEI from The Netherlands.

### 3.3. Preparation of RCDs

Firstly, 10 mg of TCPP, 1.0 g of thiourea, and 10 mL DMF were transferred into a 25 mL poly (tetrafluoroethylene)-lined autoclave. After being heated at 180 °C for 4 h, the resulting solution was slowly cooled to room temperature. The supernatant was obtained for dialysis (MWCO 3500) for 24 h and then freeze-dried to obtain the desired product.

### 3.4. Calculation of Fluorescence Quantum Yield (QY) of RCDs

The QY of RCDs was calculated through the following equation using Rhodamine B (QY = 89%) as a standard,

Φ_x_ = Φ_s_ (n_x_/n_s_)^2^ (A_s_/A_x_) (F_x_/F_s_) (1)

where “Φ” represents the QY, and “n” represents the refractive index of the solvent. The subscript “s” refers to the standard with known QY, and “x” represents the test sample.

### 3.5. Selectivity and Quantitative Analysis of Hg^2+^ and GSH

The detection of Hg^2+^ and GSH was performed at room temperature using RCDs as sensors. To evaluate the sensitivity of RCDs towards Hg^2+^, different concentrations of metal ions were added into the aqueous solution of RCDs, and the mixed solution was sonicated for 1 min before spectral measurements. The final concentration of metal ions ranged from 0 to 183 μM. To evaluate the sensitivity of RCDs-Hg (II) towards GSH, different concentration of GSH was added into the aqueous solution of RCDs-Hg (II), and the mixed solution was sonicated for 1 min before spectral measurements. The final concentration of GSH ranged from 0 to 65 µM. A similar procedure was performed for the detection of other amino acids and anions.

### 3.6. Cytotoxicity Assay

The biocompatibility of RCDs was evaluated by the MTT method against Hepa 1-6 cells. Firstly, Hepa 1-6 cells were seeded in a 96-well plate, and the corresponding medium containing 10% fetal bovine serum was added to adjust the cell density to 1 × 10^5^/well. After 24 h incubation, DMEM (200 μL) containing RCDs at different concentration (0, 3, 6, 9, 12, 15 and 18 μg/mL) was added to the designated wells. All these plates were then incubated at 37 °C for another 24 h in the dark. The sample wells were then washed twice with PBS buffer followed by the addition of 100 μL of MTT solution (0.5 mg/mL) to each well. After 4 h of incubation, the medium was removed, and the optical density (OD) at 495 nm was recorded with a microplate reader.

### 3.7. Bioimaging Test

The uptake and localization of RCDs in Hepa 1-6 cells were observed through the confocal laser scanning microscope. Firstly, Hepa 1-6 cells were incubated in an incubator at 37 °C for 24 h. After incubation with RCDs (15 μg/mL) for 10 h, the cells were washed three times with PBS buffer, and the fluorescence images were performed by a laser scanning confocal microscope.

For the localization test, Hepa 1-6 cells were incubated with RCDs at 37 °C for 12 h and then incubated with Hoechst 33,342 (50 nM) for 10 min. After washing three times with PBS solution, the cells were observed using a laser scanning confocal microscope.

### 3.8. Statistical Analysis

The statistical analysis was carried out using the IBM SPSS statistics 24.0 software. Data from both species were subjected to ANOVA (Dunnett’s multiple comparisons test). All values were presented as mean ± SD. Data were compared between the indicated groups using a *t*-test and * *p* < 0.05 was considered statistically significant.

## 4. Conclusions

In summary, the red-emissive carbon dots (RCDs) were prepared by a simple hydrothermal method with tetra (4-carboxyphenyl) porphyrin (TCPP) as a precursor. The structure and morphology of RCDs were characterized by TEM, XRD, and XPS. Compared to TCPP, RCDs exhibited better water-solubility and higher fluorescence quantum yield. Remarkably, the addition of Hg^2+^ was able to quench the FL of RCDs, while the following added glutathione can completely recover their FL intensity. Thus, RCDs could be acted as excellent FL probes for the detection of Hg^2+^ and GSH with high selectivity and sensitivity. The detection limits of Hg^2+^ and GSH based on RCDs were calculated to be 1.73 nM and 1.6 nM, respectively. Moreover, the fluorescence intensity of RCDs could return to the original level after multiple cycles, suggesting the high stability of RCDs in this sensing system. The biocompatibility and bioimaging application of RCDs were verified through the MTT method and laser scanning confocal microscope, respectively. Thus, this work provided a facile strategy to design and fabricate the highly emissive RCDs for the detection of Hg^2+^ and GSH in aqueous solutions.

## Supplementary Materials

The supporting information can be downloaded at: https://www.mdpi.com/article/10.3390/ijms23169213/s1.
