# Supplementary material for: Red-Emissive Sulfur-Doped Carbon Dots for Selective and Sensitive Detection of Mercury (II) Ion and Glutathione
Source: Int J Mol Sci. 2022 Aug 17;23(16):9213. doi: 10.3390/ijms23169213 (PMC9409242; doi:10.3390/ijms23169213)
Supplement: Supplementary file 1 [file ijms-23-09213-s001.zip › ijms-1831750-SI.pdf]

Supporting Information

Red-Emissive Sulfur-Doped Carbon Dots for Selective and Sensitive Detection of Mercury (II) Ion and Glutathione

Jinjin Zeng[1,3], Linhong Liao[1], Xiao Lin[1], Genyan Liu[1], Xiaogang Luo[1,4], Ming Luo[2], Fengshou Wu[1,3*]

1 Hubei key Laboratory of Novel Reactor and Green Chemical Technology, School of Chemical Engineering and Pharmacy, Wuhan Institute of Technology, Wuhan, 430072 P. R. China.

2 School of Materials Science and Engineering, Wuhan University of Technology, Wuhan, 430070 P. R. China.

3 Key Laboratory of Novel Biomass-Based Environmental and Energy Materials in Petroleum and Chemical Industry, Key Laboratory for Green Chemical Process of Ministry of Education, Wuhan Institute of Technology, Wuhan, 430072 P. R. China.

4 School of Materials Science and Engineering, Zhengzhou University, Zhengzhou, 450001, P. R. China.

*Corresponding authors: fswu@wit.edu.cn (F. S. Wu)

Keywords: carbon dots; red emission; Hg^{2+}; glutathione; bioimaging

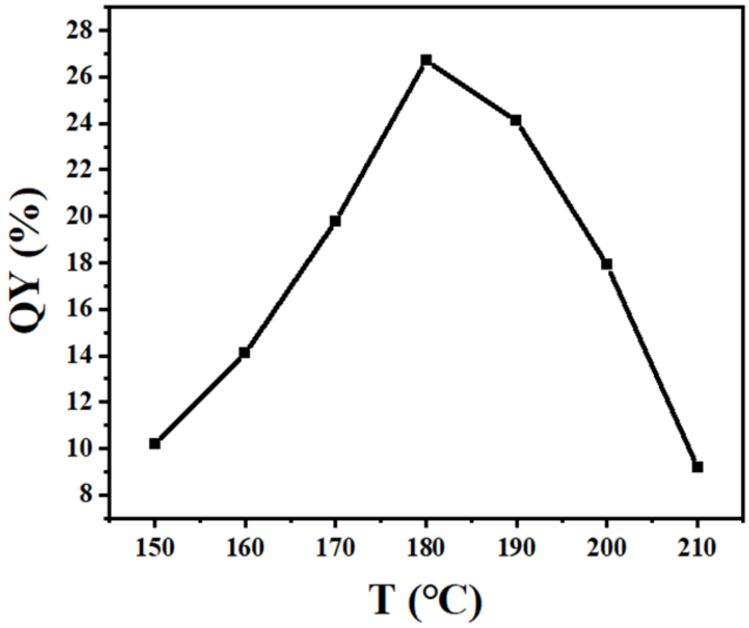

Figure S1. The fluorescence QY of RCDs prepared at different reaction temperatures.

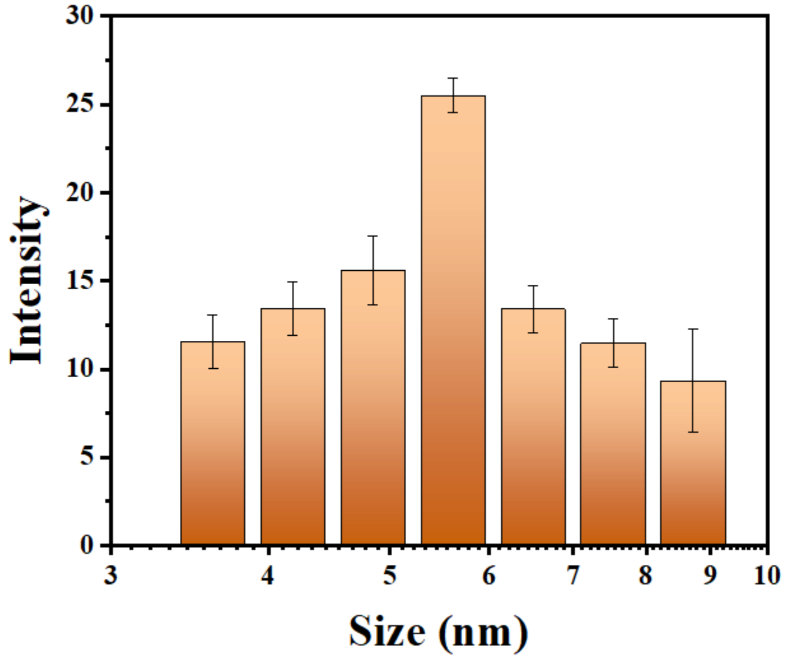

Figure S2. DLS profile of RCDs in aqueous solution

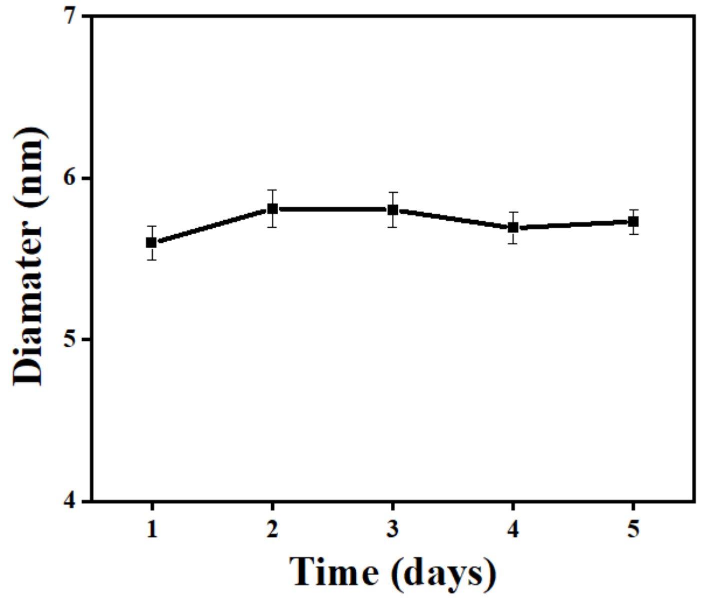

Figure S3. The DLS stability of RCDs in aqueous solution for 5 days

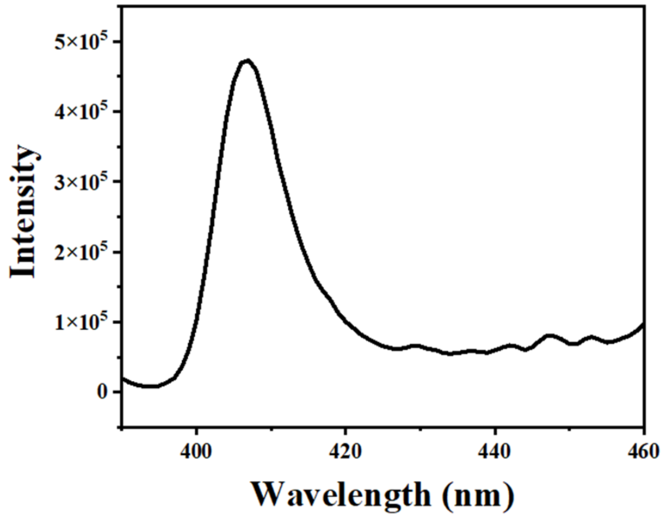

Figure S4. The excitation spectrum of RCDs (λ_{em} =650 nm) in aqueous solution
